# Risk of Stillbirth in Women with Gestational Diabetes and High Blood Pressure

**Published:** 2020-04

**Authors:** Hamid Reza TABATABAEE, Atefeh ZAHEDI, Koorosh ETEMAD, Tannaz VALADBEIGI, Sepideh MAHDAVI, Mostafa ENAYATRAD, Seideh Zeinab ALMASI, Halimeh YAGHOOBI, Fatemeh ZOLFIZADEH, Mahmoud HAJIPOUR

**Affiliations:** 1. Research Center for Health Sciences, Department of Epidemiology, School of Health, Shiraz University of Medical Sciences, Shiraz, Iran; 2. Department of Public Health, School of Health, Abadan School of Medical Sciences, Abadan, Iran; 3. Department of Epidemiology, Environmental and Occupational Hazards Control Research Center, School of Public Health, Shahid Beheshti University of Medical Sciences, Tehran, Iran; 4. Clinical Research Development Unit, Imam Hossein Hospital, Shahroud University of Medical Sciences, Shahroud, Iran; 5. Department of Epidemiology, School of Public Health, Shahroud University of Medical Sciences, Shahroud, Iran; 6. Department of Epidemiology, School of Medicine, Dezful University of Medical Sciences, Dezful, Iran; 7. Health Research Center, Zahedan University of Medical Sciences, Zahedan, Iran; 8. Mother and Child Welfare Research Center, Hormozgan University of Medical Sciences, Bandar Abbas, Iran; 9. Pediatric Gastroenterology, Hepatology, and Nutrition Research Center, Research Institute for Children’s Health, Shahid Beheshti University of Medical Sciences, Tehran, Iran

**Keywords:** Stillbirth, Gestational diabetes, Hypertension, Iran

## Abstract

**Background:**

Both Gestational diabetes and hypertension almost affect 10.5% of the pregnancies. This study was conducted to investigate and compare the pregnancy outcomes in women with gestational diabetes or high blood pressure with outcomes belonging to healthy mothers.

**Methods:**

This population-based case-control study was conducted in 8 provinces and two cities of Iran on women referred to the public health centers during 2015 to 2018. Descriptive statistics for variables presented by percentages and frequencies and logistic regression analysis was used to analyze data at a significance level of less than 0.05.

**Results:**

Some variables such as ethnicity, maternal education and age, gestational diabetes, high blood pressure and previous pregnancy outcome were significantly associated with stillbirth. Maternal age greater than 35 yr (OR=1.78, CI: 1.29–2.48), maternal illiteracy (OR=3.67, CI: 2.25–5.98), a previous stillbirth (OR=9.92, CI: 4.98–19.78), gestational diabetes among women who had never had a screening test (OR =3.91, CI: 2.96–5.18) and high blood pressure (OR =1.95, CI: 1.38–2.77) were important factors associated with stillbirth. Maternal and paternal occupation, paternal education and age, place of residence, smoking and maternal BMI were significantly associated with stillbirth.

**Conclusion:**

Gestational diabetes, hypertension, a previous miscarriage, stillbirth, first pregnancy, low education level, advanced maternal age and ethnicity were associated with an increased risk of stillbirth. It is necessary to provide high-quality healthcare services before and during pregnancy particularly for those at heightened risk and improve knowledge of mothers on the side effects of each of the mentioned risk factors in order to control these factors more effectively and thus reducing the risk of stillbirth.

## Introduction

Diabetes is one of the most common health problems with two variations, type 1 diabetes most frequently occurs in young people and it is associated with deficiency (or lack) of insulin. Type 2 diabetes is linked with relative insulin deficiency and insulin resistance which often occurs in overweight and inactive people (1). Diabetes can surface during pregnancy, known as Gestational Diabetes and can be defined as impaired glucose tolerance (1, 2) first being diagnosed (or beginning) during pregnancy (2). In addition to diabetes, high blood pressure is also a common medical condition during pregnancy. High blood pressure is the second leading cause of maternal mortality worldwide (3).

Women with diabetes before pregnancy face an increased risk of infant mortality and having a diabetic baby. In England, diabetic mothers (type 1 and 2) were 5 times more likely to have stillbirths compared with non-diabetic mothers. Moreover, the chance of infant mortality during the first 3 months of life among these mothers was 3 times higher (4). In developed countries, the incidence of gestational diabetes is 5%–10% (5). Hypertension in pregnancy may be associated with risk of adverse outcomes such as stillbirth, premature birth, Intrauterine Growth Retardation (6). The effects of hypertension on the risk of fetal death have not been fully investigated. The risk of fetal death varies throughout pregnancy and it increases after week 37 of pregnancy. On the one hand, prevalence of hypertension in pregnant women increases with increasing gestational age; therefore, increased risk of fetal death in the perinatal period can be partly linked to hypertension (7). Gestational diabetes occurs approximately in 2%–5% of all pregnancies, which varies from 1%–14% in different countries (2). hypertension disorder also affects 5%–8% of pregnancies (8).

Since there are several risk factors for stillbirth, identifying these factors in order to carry out preventive measures in each geographic region is necessary while no comprehensive account of this issue was available in Iran, this study was conducted to investigate the risk factors for stillbirth.

## Materials and Methods

This study was a population-based case-control method on mothers who visit primary health care (PHC) units in Iran. Case group includes mothers who had stillbirth in last pregnancy. In the period 2015–2018, data were collected from public health care centers in 8 provinces and 2 cities (Fars, Hormozgan, Kermanshah, Hamedan, Kohgilueh-and-Boyerahmad, Yazd, South Khorasan, and Golestan provinces and Mashhad and Zahedan cities). In any province, capital city and four other towns (separately from north, west, east and south of province) were selected. From PHC units of selected cities and towns, an urban and a rural unit were selected and questionnaire was distributed and data was collected.

Ethical approval was granted by Shiraz university of Medical Sciences Research Ethics Committee (Ir.sums.rec.1394.f330). All participants provided written informed consent prior to interview; this included permission to use anonymous quotations in publications.

Information of stillbirth collected using survival sheet. Survival sheet is a simple and effective tool of PHC information system that is used in PHC units. It is a sheet with 70 cm in length and 50 cm in width that is used just to represent birth and death. Information that is recorded on survival sheet is compared to hospital information and mistakes are corrected. Therefore, recorded information on survival sheet is very precise. Using following formula and considering the risk of mothers with age more than 35 and considering design effect on cluster sampling, number of samples in any groups was estimated 1050 mother. In this investigation, with aid of PHC units and in order to increase precision of study and decreasing random error, information of 3080 mother was collected. Overall, 1458 mothers have stillbirth in their last pregnancy (cases) and 1626 mothers with children born healthy; so, they considered as control group.

Data gathering tool in this investigation was a questionnaire which includes three sections. First section was parent’s information (including education, mother’s job, working of mother at night shift, father’s job, race and place of residence), second one were conditions at former pregnancy and last one was baby status (weight, gender, etc.).

Considering that experts of PHC units are first line of health caring in Iran, questionnaire are distributed between them. Then, information evaluation was done with same regulation and with investigation of files of contributors. Questionnaire verified with experts and its reliability examined with Cronbach’s alpha of 0.66 in a pilot study on 50 mothers in Kermanshah Province. Collected data was interred in Excel software, firstly. After data correction and cleaning the data for uncompleted forms, data was interred in SPSS software. Analysis was done in this software using Chi-Square test and binary logistic regression.

## Results

Overall, 1458 mothers as case group, and 1626 mothers, as control group were selected. The majority of the participants (91%) were below 35 yr of age. 36.5% of the mothers had high school education and 90.8% were housewives.

Table 1 presents the information pertaining to the relationship between the demographic variables and stillbirth. There was no significant relationship found between the stillbirths and place of living and ethnicity in univariate analysis. However, other variables such as mother’s and father’s age, education, history of stillbirth and abortion, mother’s occupation (working on night shift) father’s occupation, GDM, hypertension and etc. related to stillbirth (Table 1). Mothers with ages above 35 yr in respect to mothers with ages below 35 yr showed a greater chance odds of stillbirth (OR=2.14(CI:1.65–2.78)). Education level was also found associated with the stillbirth in such a manner that the more the individuals were found having higher education degrees the lesser the stillbirth rates were. In comparison to the mothers who had university education, illiterate mothers had 3.39 times higher chances odds of stillbirth and this was 2.68 times for mothers with primary school schooling education, 2.23 times for mothers with secondary school education and 1.56 times for mothers with high school education.

**Table 1:** Frequency Distribution of Demographic Variables in Case and Control Groups

***Variables***		***Case N(%)***	***Control N(%)***	***Total N(%)***
Place	Urbane	658(46.4)	761(53.6)	1419(47.2)
	Rural	760(47.9)	825(52.1)	1585(52.8)
Maternal age	≤ 35	1257(45.4)	1514(54.6)	2771(91.1)
	35 <	173(64.1)	97(35.9)	270(8.9)
Paternal age	≤ 37	1166(45.6)	1390(54.4)	2556(84.3)
	37 <	259(54.4)	217(45.6)	476(15.7)
Ethnic	Fars	830(44.9)	1019(55.1)	1849(61.3)
	Lur	93(46.3)	108(53.7)	201(6.7)
	Turkish	257(50.7)	250(49.3)	507(16.8)
	Kurd	34(68)	16(32)	50(1.7)
	Arab	19(54.3)	16(45.7)	35(1.2)
	Baluch	99(49.5)	101(50.5)	200(6.6)
	Turkmen	74(54.4)	62(45.6)	136(4.5)
	Other	15(37.5)	25(62.5)	40(1.3)
Maternal education	Illiterate	108(7.4)	68(38.6)	176(5.7)
	Primary school	395(55.6)	315(44.4)	710(23.1)
	Middle school	363(51.1)	347(48.9)	710(23.1)
	High school	475(42.3)	648(57.7)	1123(36.5)
	University graduate	115(31.9)	246(68.1)	361(11.7)
Previous pregnancy outcome	A live birth	713(43)	947(57)	1660(55.7)
	Stillbirth	89(86.4)	14(13.6)	103(3.5)
	Miscarriage	123(61.8)	76(38.2)	199(6.7)
	First pregnancy	484(747.7)	530(52.3)	1014(34)
	Other	2(40)	3(60)	5(0.2)
Smoking during Pregnancy	No	72(5)	83(5.2)	155(5.1)
	Yes	1362(95)	1525(94.8)	2887(94.9)
BMI	<20	680(51.6)	821(54.8)	1501(53.3)
	20 – 24.9	224(17)	242(16.2)	466(16.5)
	25 – 29.9	304(23)	323(21.6)	627(22.3)
	30 – 34.9	82(6.2)	84(5.6)	166(5.9)
	35 – 40	29(2.2)	27(1.8)	56(2)
Maternal Working at Night Shifts	Yes	15(32.6)	31(67.4)	46(1.67)
	No	1315(48.5)	1394(51.5)	2709(98.33)
Paternal Education	Illiterate	69(57)	52(43)	121(3.9)
	Primary school	290(55.1)	236(44.9)	526(17.1)
	Middle school	471(52)	434(48)	905(29.4)
	High school	475(42.5)	642(57.5)	1117(36.3)
	University graduate	150(36.7)	259(63.3)	409(13.3)
Maternal Occupation	Housewife	1339(48.3)	1433(51.7)	2772(90.8)
	Employee	65(32.5)	135(67.5)	200(6.6)
	Farmer, Herder, Carpet Weaver Other	25(56.8)	19(43.2)	44(1.4)
		18(48.6)	19(51.4)	37(1.2)
Paternal Occupation	Self-employed	937(58.5)	993(51.5)	1930(63.1)
	Employee	178(37.4)	298(62.6)	476(15.6)
	Farmer	104(49.1)	108(50.9)	212(6.9)
	Herder	30(53.6)	26(46.4)	56(1.8)
		201(52.1)	185(47.9)	386(12.6)
Hypertension	No	1326(90.9)	1547(95.1)	2873(93.2)
	Yes	132(9)	79(4.9)	211(6.8)
^*^Gestational Diabetes	Group 1	999(68.5)	1353(83.2)	2352(79.2)
	Group 2	90(6.2)	79(4.9)	169(5.7)
	Group 3	21(1.4)	20(1.2)	41(1.4)
	Group 4	289(19.8)	120(7.4)	409(13.8)

* Group 1 included women without any GDM; Group 2 included women with GDM who visited PHC units; Group 3 included women with impaired glucose given only a diet with no need to visit a physician; Group 4 included women not do any tests.

The results of the prior pregnancies were also found associated with the stillbirth in one-variable analyses. The individuals whose prior pregnancies had ended in stillbirth, as compared to the individuals with normal delivery, had an elevated chance of stillbirth for 8.44 (CI:4.76–14.95) times and this was reduced to 2.15 (CI:1.58–2.90) times in individuals had abortions as well as 1.21 (CI:1.03–1.41) times that was scored for the ones giving their first birth. The women whose spouses were employees versus the ones married to free-lancers demonstrated 37% (OR=0.63, CI: 0.51–0.77) lower chance of stillbirth. This is while no significant relationship was observed in occupations such as agriculture, ranching and other jobs. Type 2 diabetes and hypertension were found in a significant relationship with stillbirth. The mothers diagnosed with gestational diabetes mellitus and had also referred to medical centers (OR=1.54, CI: 1.12–2.11) and the mothers not undergone a check-up (OR=3.26, CI: 2.59–4.09) had a higher chance for stillbirth in comparison to the mothers not diagnosed with gestational diabetes mellitus. In the meantime, the mothers who had blood glucose irregularities and had only received nutritional regimes not showed a significant relationship with stillbirth (OR=1.42, CI: 0.76–2.63). Mother’s smoking and body mass index were not found in had not any significant relationship with stillbirth. Variables exhibiting significant association in univariate analysis were further evaluated by means of multivariate binary logistic regression. The odds ratios and the adjusted confidence limits are summarized in Table 2.

**Table 2:** Univariate and Multivariate logistic regression risk of stillbirth

***Variables***		***Or (%95 ci)***	***P-value***	***Or_adj_(%95 ci)***	**P-*value***
Place	Urbane	Reference	Reference	-	-
	Rural				
Maternal age	≤ 35	Reference	Reference	Reference	Reference
	35 <				
Paternal age	≤ 35	Reference	Reference	-	-
	35 <				
Ethnic	Fars	Reference	Reference	Reference	Reference
	Lur				
	Turkish				
	Kurd				
	Arab				
	Baluch				
	Turkmen				
	Other				
Maternal education	Illiterate	3.39(2.33–4.94)	0.001	3.67(2.25–5.98)	0.001
	primary school	2.68(2.05–3.5)	0.001	3.2(2.28–4.48)	0.001
	middle school	2.23(1.71–2.91)	0.001	2.94(2.11–4.1)	0.001
	high school	1.56(1.22–2.01)	0.001	2.04(1.5–2.79)	0.001
	university graduate	Reference	Reference	Reference	Reference
Previous pregnancy outcome	A live birth	Reference	Reference	Reference	Reference
	Stillbirth				
	Miscarriage				
	first pregnancy				
	Other				
Maternal working at night shifts	No	0.51(0.27–0.95)	0.035		
	Yes	Reference	Reference	-	-
Paternal education	Illiterate	2.29(1.51–3.46)	0.001	-	-
	primary school	2.12(1.62–2.76)	0.001		
	middle school	1.87(1.47–2.38)	0.001		
	high school	1.27(1.01–1.61)	0.04		
	university graduate	Reference	Reference		
Maternal occupation	Housewife	Reference	Reference	-	-
	Employee	0.51(0.38–0.69)	0.001		
	Farmer, herder, carpet	1.4(0.77–2.56)	0.264		
	weaver	1.01(0.53–1.94)	0.967		
Paternal occupation	Self-employed	Reference	Reference	-	-
	Employee	0.63(0.51–0.77)	0.001		
	Farmer	1.02(0.76–1.35)	0.888		
	Herder	1.22(0.71–2.08)	0.459		
	Other	1.15(0.92–1.43)	0.206		
Hypertension	No	Reference	Reference	Reference	Reference
	Yes	1.94(1.46–2.6)	0.001	1.95(1.38–2.77)	0.001
^*^gestational diabetes	Group 1	Reference	Reference	Reference	Reference
	Group 2	1.54(1.12–2.11)	0.007	1.49(1.02–2.17)	0.030
	Group 3	1.42(0.76–2.63)	0.260	0.78(0.37–1.65)	0.520
	Group 4	3.26(2.59–4.09)	0.001	3.91(2.96–5.18)	0.001

* Group 1 included women without any GDM; Group 2 included women with GDM who visited PHC units; Group 3 included women with impaired glucose given only a diet with no need to visit a physician; Group 4 included women not do any tests.

An inverse relationship was observed between stillbirth and mother’s education in such a manner that the odds of stillbirth was found lowering with an elevation in mother’s education. In comparison to the mothers who had university education, illiterate mothers showed a 3.67 (CI: 2.25–5.98) times higher stillbirth chance and this value was 3.2 (CI: 2.28–4.48) times for mothers who had primary school education as well as a 2.94 (CI: 2.11–4.1) times value for mothers having secondary school education and 2.04 (CI: 1.5–2.79) times for mothers with high school education. Stillbirth was higher in Turk ethnicities (OR=1.37,CI: 1.09–1.72) in comparison to Fars ethnicities and it was also found lesser in Baluch ethnicity (OR=0.5,CI: 0.33–0.75) in contrast to the Fars ethnicities. Mothers who had a past history of dead child (OR=9.92,CI:4.98–19.78), mothers with abortions (OR=1.93,CI: 1.34–2.77) and mothers in their first pregnancy (OR=1.37,CI: 1.14–1.65) indicated a higher chance of stillbirth in comparison to mothers who had given birth to their normal children in their previous delivery.

Mothers with hypertension showed a stillbirth chance of almost twice the healthy mothers (OR=1.95, CI: 1.38–2.77). Type II diabetes was not found to be statistically significant in multivariate analyses. Mothers with diabetes referred to medical centers (OR=1.49, CI: 1.02–2.17) and mothers not undergone check-ups (OR=3.91, CI: 2.96–5.18) showed higher chances of stillbirth in comparison to the mothers not diagnosed with gestational diabetes mellitus at all. However, the mothers not diagnosed with blood glucose abnormalities and had, therefore, only received dietary regimes (OR=0.78, CI: 0.37–1.65) did not indicate significant difference from the healthy mothers.

## Discussion

The relationship between high blood pressure, diabetes and some individual variables with the birth of a baby were investigated in order to evaluate the risk factors of neonates in Iran. The relationship between maternal age (over 35 yr), the ethnic group to leave, mothers with low education and date of birth and abortions in previous pregnancies, first pregnancy, gestational diabetes and high blood pressure and birth were observed. Advanced maternal age was significantly associated with the risk of still, this relationship remained significant even after taking into account the factors linked with increased age such as diabetes, high blood pressure and multiple pregnancies. These results are consistent with some other studies (9). In Mexico, being younger than 18 and older than 34 yr was associated with an increased risk of stillbirth (10) while in some other studies the relationship between maternal age and stillbirth was not confirmed (11, 12). Increased maternal age increases the risk of preterm birth or congenital anomaly; however, by using new diagnostic methods such cases have decreased in areas wherein these methods are available (13). The paternal age was also investigated in this study which by considering the effects of other variables, was not significantly associated with stillbirth.

In our research, education, occupation and night work were considered as protective factors in a uniform analysis; however, considering the effects of other variables, the only remaining variable was education. In other studies, higher education was associated with a reduction in maternal mortality (10), while mothers were more likely to be pregnant compared to housewives (12). In fact, the observed relationship can be related to the level of education.

Studies on ethnicity and risk of stillbirth showed that ethnic minority was a risk factor for stillbirth (12) while Asian race was a protective factor (9). In this study, due to existence of various ethnic groups in Iran, the relationship between this factor and stillbirth was examined and the results showed an increased risk of stillbirth in Turk ethnic group and reduced risk of stillbirth among Baluch ethnic group compared with Fars ethnic group. Sistan and Baluchestan Province ranked last among provinces of Iran in terms of access to health indicators (14) Therefore, there is the possibility of having home deliveries and failure to register them which leads to under-reporting of stillbirths in this region.

Paternal education and occupation were also the factors associated with stillbirth in univariate analysis. The risk of stillbirth among the mothers whose husbands had higher education levels and were employees was lower associated with financial status of the family and their access to medical care services. This association was observed in a study (12); however, in our study this relationship became insignificant by considering other variables.

In some studies, smoking is a risk factor for stillbirth (15); however, such association was not observed in our study. On the one hand, this inconsistency may be due to the low number of female smokers in Iran and on the other hand, people refrain from telling the truth because women smoking cigarette is not accepted in our social culture and has no positive social image.

There is no relationship between the place of residence and the birth of the baby in both multivariate analysis and multivariate analysis, which indicates that the quality of care during pregnancy in the villages is as large as cities and there is no difference between them. In some studies, body mass gain during pregnancy is associated with an increased risk of preeclampsia resulting in stillbirth (16, 17) while this relationship was not significant in our study. This inconstancy is probably due to this fact that we did not directly take accurate height and weight measurements and calculated the index based on the individuals’ reports.

In this study, those previously experienced a stillbirth, miscarriage or even women who were pregnant for the first time were at greater risk. This association remained significant after considering the effects of other variables among which having a history of stillbirth was associated with a much higher risk. In some studies, this relationship was not observed (12, 18) while the relationship between the first pregnancy and stillbirth was approved in a cohort study (12).

The relationship between gestational diabetes and stillbirth was investigated in many of the studies. In this study, in order to examine the impact of gestational diabetes more accurately the participants we divided into 4 groups so that we could separately examine the impact of each group (Table 1). In univariate analysis, those with gestational diabetes referred, people with impaired glucose tolerance who did not require a referral and just received a diet and those never done a test had a greater chance of stillbirth compared with those without gestational diabetes. After considering the effects of other variables, the group with impaired glucose tolerance who they did not need any treatments did not remain in the model while the other two groups (people with diabetes referred and those never tested) had a great chance for stillbirth; whereas those never tested had a higher chance of stillbirth. It clearly highlights the need for taking pregnancy tests and receiving a proper prenatal care. Some other studies have reported similar results as well (19, 20) while some other had different results (12, 15). This inconsistency of the results can be linked to the sample size and the plan. On the other hand, in developed countries prenatal care is being performed attentively especially for those who need special care because of experiencing a high risk pregnancy and it may help reduce the complications among these patients.

In this study, hypertension was considered as an influential factor for stillbirth and this fact was also confirmed by other studies (6, 8, 9). From the second birth onwards, the complications of hypertension may be more severe during pregnancy which may be due to an increased rate of chronic diseases (e.g. diabetes) and obesity by increasing maternal age. Weight gain and obesity both before getting pregnant and during pregnancy are associated with increased blood pressure during pregnancy. Since the chance of obesity and diabetes is higher among multiparous women, more severe preeclampsia occurs among this group (8). Recently the age of mothers giving birth to their first child has increased among Iranian women (21) and additionally, increasing the birthrate lies at the heart of the country’s policies, blood pressure, obesity, and diabetes are on the rise which are the risk factors for developing preeclampsia in pregnant women resulting in some complications such as an increased rate of neonatal mortality and stillbirth among pregnant women. Therefore, regular monitoring of blood pressure during pregnancy is necessary and it is highly emphasized.

The advantages of this study include its large sample size and sampling from various provinces of the country which makes the results general-izable to the whole population of Iran. The comprehensive investigation of the factors associated with stillbirth and neonatal mortality can be considered as other advantages of this study. One of the disadvantages of this study is using information of health records and given that this information was not for research purposes some required information such as smoking by mother was incomplete and the respondents refused to answer related questions because it contradicted our society culture.

## Conclusion

Gestational diabetes, hypertension, ethnicity, low education level, maternal age and a history of miscarriage, stillbirth and first pregnancy were found associated with an increased risk of stillbirth; whereas low education, a history of stillbirth in previous pregnancy and developing gestational diabetes (without any diagnostic tests) were the major risk factors in our study. These factors highlight the need to take necessary measures in order to elevate mothers’ knowledge about receiving proper care both before pregnancy and during pregnancy, explain them the side effects of each of the mentioned risk factors and improve the level of services across the health care centers.

## Ethical considerations

Ethical issues (Including plagiarism, informed consent, misconduct, data fabrication and/or falsification, double publication and/or submission, redundancy, etc.) have been completely observed by the authors.
